# Incorporating the inflammation-related parameters enhances the performance of the nomogram for predicting local control in lung cancer patients treated with stereotactic body radiation therapy

**DOI:** 10.1007/s00432-024-05811-5

**Published:** 2024-05-29

**Authors:** Bao-Tian Huang, Pei-Xian Lin, Li-Mei Luo, Ying Wang

**Affiliations:** 1 Department of Radiation Oncology, Cancer Hospital of Shantou University Medical College, Shantou, 515031 Guangdong China; 2 Department of Nosocomial Infection Management, The Second Affiliated Hospital of Shantou University Medical College, Shantou, 515041 Guangdong China; 3 Department of Radiation Oncology, Affiliated Cancer Hospital & Institute of Guangzhou Medical University, Guangzhou, 510095 Guangdong China

**Keywords:** Inflammation-related parameters, Nomogram, Local control, Lung cancer, Stereotactic body radiation therapy

## Abstract

**Purpose:**

The study aims to investigate whether including the inflammation-related parameters would enhance the accuracy of a nomogram for local control (LC) prediction in lung cancer patients undergoing stereotactic body radiation therapy (SBRT).

**Methods:**

158 primary or metastatic lung cancer patients treated with SBRT were retrospectively analyzed. The clinical, dosimetric and inflammation-related parameters were collected for the Cox regression analysis. The ACPB model was constructed by employing the clinical and dosimetric factors. And the ACPBLN model was established by adding the inflammation-related factors to the ACPB model. The two models were compared in terms of ROC, Akaike Information Criterion (AIC), C-index, time-dependent AUC, continuous net reclassification index (NRI), integrated discrimination improvement (IDI), calibration plots and decision curve analysis (DCA).

**Results:**

Multivariate Cox regression analysis revealed that six prognostic factors were independently associated with LC, including age, clinical stage, planning target volume (PTV) volume, BED of the prescribed dose (BEDPD), the lymphocyte count and neutrocyte count. The ACPBLN model performed better in AIC, bootstrap-corrected C-index, time-dependent AUC, NRI and IDI than the ACPB model. The calibration plots showed good consistency between the probabilities and observed values in the two models. The DCA curves showed that the ACPBLN nomogram had higher overall net benefit than the ACPB model across a majority of threshold probabilities.

**Conclusion:**

The inflammation-related parameters were associated with LC for lung cancer patients treated with SBRT. The inclusion of the inflammation-related parameters improved the predictive performance of the nomogram for LC prediction.

## Introduction

Lung cancer is the leading cause of cancer-related death worldwide (Leiter et al. 2023). Stereotactic body radiation therapy (SBRT) has been widely adopted as an effective and well-tolerated treatment for medically inoperable patients with early stage non-small cell lung cancer (NSCLC) (Andruska et al. 2021). This technique delivers a higher radiation dose to the target in a few fractions, resulting in a high biologically effective dose (BED). The goal of the technique is to achieve maximum therapeutic efficacy while minimizing the impact on organs at risk (OARs) (Brown et al. 2014; Moreno et al. 2020). Despite encouraging outcomes, some patients still experience local recurrence, with recurrence rate of 13.0% and 18.8% after SBRT (Lee et al. 2022; Kim et al. 2020). Therefore, it is important to develop a model for predicting local recurrence in lung cancer patients treated with SBRT to determine more personalized treatment strategies.

The primary strategy for local control (LC) prediction is the use of radiobiological model in which the dosimetric and other clinical factors are employed to establish the dose–response relationship using mathematical formula. However, this method fails to consider inflammation-related factors, which were reported to be correlated with prognosis in lung cancer patients treated with SBRT (Cannon et al. 2015; Dong et al. 2023; Kotha et al. 2021; Huang et al. 2023; Sebastian et al. 2019; Aduquaye et al. 2022). Several independent studies have reported the relationship between inflammation-related factors and overall survival (OS) after SBRT in lung cancer (Dong et al. 2023; Kotha et al. 2021; Huang et al. 2023; Sebastian et al. 2019). However, there is limited evidence regarding the prognostic value of inflammation-related factors specifically for local recurrence. Therefore, further investigation is needed to determine the potential prognostic value of these easily accessible and low-cost inflammatory biomarkers for LC prediction. It is also unclear whether incorporating these factors into prediction models can improve the precision and accuracy of prognostic predictions.

Therefore, the aim of our study is two-fold: (1) To clarify whether the inflammation-related factors are associated with LC for lung cancer patients treated with SBRT. (2) To explore whether inclusion of these factors will improve the accuracy of the nomogram.

## Methods

### Study population

The study included 189 patients who were diagnosed with primary or secondary lung cancer and treated with SBRT at the Cancer Hospital of Shantou University Medical College between July 2011 and April 2021. To be included, patients had to meet the following criteria: (1) confirmed diagnosis of primary or secondary lung cancer treated with SBRT; (2) complete baseline clinical information and follow-up data available for all patients; (3) nominal BED of ≥ 70 Gy_10_.

### Treatment

Risk-adapted dose schedules were utilized for treatment, considering factors such as tumor size and proximity to OARs. Commonly used dose schedules included 12.5 Gy × 4, 25 Gy × 1, 10.0 Gy × 5, 10.0 Gy × 4 and 12.0 Gy × 4. For example, the 12.5 Gy × 4 schedule referred to delivering 50 Gy in 4 fractions, with similar definitions for the other schedules. The interval target volume (ITV) of the tumor was obtained by combining ten phases of the moving tumor generated from the four-dimensional computed tomography (4DCT). To ensure that the radiation therapy adequately covers the tumor, a planning target volume (PTV) was generated by uniformly expanding the ITV in all directions by 5 mm. The treatment planning was performed on the average image projection of the 4DCT, with the PTV serving as the target volume. Treatment planning was conducted using the Eclipse treatment planning system (with Version 10.0 used from July 2011 to September 2019, and Version 15.5 from October 2019 to April 2021, Varian Medical System, Inc., Palo Alto, CA). All patients were treated on a TrueBeam LINAC with RapidArc or intensity-modulated radiotherapy (IMRT) technique. Prior to each treatment fraction, cone beam computed tomography (CBCT) scanning was performed to correct for setup errors. Additionally, for some patients, tumor motions were evaluated beforehand using the fluoroscopic scanning capabilities of the LINAC to ensure that the tumor remained within the PTV.

### Follow-up

The patients underwent CT scans every 3 months in the first year after treatment. Afterwards, the frequency of evaluations was reduced to every 6 months (Schneider et al. 2018). The latest follow-up was conducted in September 2021. Tumor LC was determined as the lack of local tumor recurrence at the treatment site. Local recurrence was determined mainly by biopsy and the progressive growth of the primary tumor or its vicinity on two consecutive CT scans with a minimum 6-month interval. Histologic confirmation was performed when local recurrence was highly suspected. However, if a biopsy specimen was not feasible, diagnosis was made through contrast enhanced CT or PET/CT. Alternatively, local recurrence diagnosed by CT scans should consider the clinical symptoms and oncologist assessments (Ohri et al. 2012; Lenglet et al. 2019).

### Data collection

Patients’ clinical characteristics were collected, including gender, age, clinical stage, primary or metastatic lung cancer, smoking status, histology, PTV volume, and maximum diameter (MD). Dosimetric parameters, such as the prescribed dose recorded as BED (BEDPD), the maximum BED in PTV (BEDPTV_max_), and treatment duration, were also collected. The BED was calculated using the linear-quadratic (LQ) model with an α/β ratio of 10 Gy (Guckenberger et al. 2016). The formula for calculating BED is BED = *n* × *d* × [1 + *d*/(α/β)], where *n* represents the fraction number and *d* represents the fractional dose. Before the radiation therapy, the inflammation-related parameters, including the lymphocyte count, neutrocyte count, platelet-to-lymphocyte ratio (PLR), neutrocyte-to-lymphocyte ratio (NLR), systemic immune-inflammation index (SII, calculated as platelet counts × neutrocyte counts ÷ lymphocyte counts), lymphocyte ratio (LR), and hemoglobin concentration, were also collected.

### Univariate and multivariate Cox regression

In this study, continuous variables were transferred into categorical ones. The optimal cut-off point were determined by X-tile (Camp et al. 2004). Prognostic factors for LC were identified using univariate Cox proportional hazards regression analysis. Variables with a significant level of *P* ≤ 0.10 in univariate analysis were further analyzed using a backward multivariate Cox regression analysis with the Akaike Information Criterion (AIC).

### Model construction

Two prediction models were constructed. The ACPB model combined the clinical and dosimetric predictors that showed significance in the multivariate Cox regression analysis. Another model was constructed to examine whether adding inflammation-related factors would enhance the accuracy of the prediction models. This model combined the inflammation-related parameters that were identified as significant at the previous multivariable Cox regression analysis with the factors from the ACPB model. The two models were compared using various metrics such as AUC value, AIC value, C-index, time-dependent AUC curves, continuous net reclassification index (NRI), integrated discrimination improvement (IDI), calibration plots, and decision curve analysis (DCA). AIC is a statistical measure used for model selection and comparison. The lower the AIC value, the better the model fits the data. The C-index ranges from 0.5 (random chance) to 1 (perfect prediction). Generally, a C-index exceeding 0.7 is considered good, and over 0.8 is excellent (Pencina and D’Agostino 2015). NRI and IDI are two statistical indexes used to compare the performance of different prediction models. These indexes are more sensitive to improvement in predictive performance than the AUC values. NRI measures the improvement in correct reclassification of individuals into relevant risk categories by comparing two prediction models. IDI calculates the average difference in predicted probabilities between the new and old models for both events and non-events cases. NRI greater than 0 indicates that the new model shows a positive improvement compared to the old model. NRI less than 0 indicates a negative improvement in the new model compared to the old model, while NRI equal to 0 indicates that there is no improvement between the new and old models. The IDI index shares a similar definition. The IDI index shares a similar definition. The calibration performance of the nomogram was evaluated using a calibration plot to assess the agreement between the predicted probability from the nomogram and the Kaplan–Meier estimate. A 45-degree calibration curve represents an ideal prognostic prediction (Kang et al. 2020). The DCA, which quantifies the net benefit at different threshold probabilities, was employed to determine the clinical utility of the model. Internal validations were performed using the bootstrapping method with 500 resamples. The bootstrap sampling method was performed on the C-index, time-dependent AUC, NRI, IDI and calibration plot. All the bootstrapping performed only in the backward multivariate Cox regression modeling.

### Statistical analysis

Statistical analyses were conducted in R software package (version 4.2.1) and PCPM (V3.22, Jingding Medical Technology Co., Ltd.). Univariate and multivariate Cox proportional hazards regression analysis was performed with the *survival* package in R. The forest plot was created using the *forestplot* package. The calibration curves were generated with the *rms* package in R. Discriminative analysis was conducted using the survivalROC function within the *survival* package. Time-dependent AUC curves were plotted using the *pec* package. The C-index comparison between the models were analyzed with the *Cschange* package. DCA curves were generated using the *stdca.R* package. AUC comparison between the two models was performed using the DeLong test. *P* < 0.05 were considered statistically significant. The NRI and IDI were calculated using the *nricens* package and *survIDINRI* package in R, respectively.

## Results

### Univariate and multivariate Cox regression

**Fig. 1:**
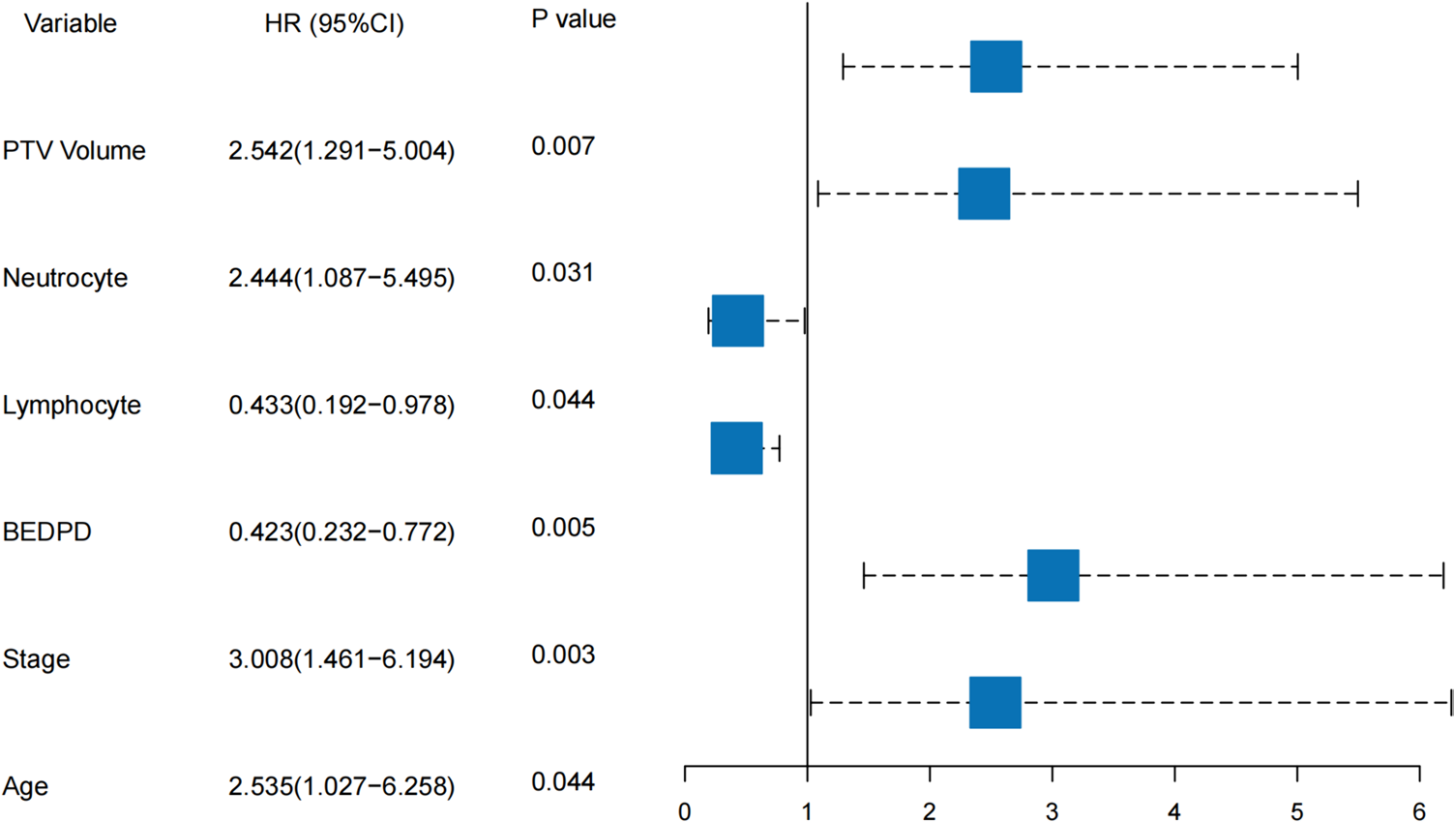
The forest plot of the multivariate analysis. PTV = planning target volume, BEDPD = prescription dose recorded as BED

158 primary or metastatic lung cancer patients treated with SBRT were retrospectively analyzed finally. Until the last follow-up, 35.4% patients (56/158) experienced local recurrence. The median follow-up time for the entire cohort was 40 months (95% CI: 34–46). In the univariate Cox analysis, the *P* values for the following factors were less than 0.1, including clinical stage, PTV volume, MD, BEDPD, BEDPTV_max_, lymphocyte count, neutrocyte count, PLR, NLR and LR. After the multivariate analysis, factors such as age, clinical stage, PTV volume, BEDPD, lymphocyte count and neutrocyte count were found to be independently associated with LC. The results of the univariate Cox analyses were presented in Table 1. The forest plot of the multivariate analysis was shown in Fig. 1.

**Table 1:** Univariate analysis results for LC

	Univariate analysis	
	*p*-Value	HR (95%CI)
Gender		
Male	0.119	Reference
Female		1.547 (0.895–2.674)
Age		
≤ 75	0.073	Reference
> 75		2.177 (0.931–5.089)
Clinical stage		
I	0.001	Reference
II ~ IV		3.332 (1.676–6.626)
Tumor origin		
Primary	0.643	Reference
Metastatic		1.133 (0.667–1.925)
Smoking status		
Non-smoker	0.486	Reference
Smoker		0.830 (0.49–1.403)
Histology		
Adenocarcinoma		Reference
SCC	0.827	0.922 (0.444–1.914)
Unknown	0.242	1.434 (0.784–2.622)
PTV volume (cc)		
≤ 119	0.001	Reference
> 119		3.027 (1.588–5.769)
MD (cm)		
≤ 5.6	< 0.001	Reference
> 5.6		3.313 (1.696–6.473)
BEDPD		
≤ 80.0	0.006	Reference
> 80.0		0.452 (0.255–0.800)
BEDPTV_max_		
≤ 114.0	0.076	Reference
> 114.0		0.620 (0.366–1.051)
Duration (days)		
≤ 3.0	0.114	Reference
> 3.0		1.895 (0.858–4.186)
Lymphocyte count (× 10^9^/L)		
≤ 2.2	0.072	Reference
> 2.2		0.482 (0.218–1.066)
Neutrocyte count (× 10^9^/L)		
≤ 2.68	0.015	Reference
> 2.68		2.547 (1.202–5.396)
PLR		
≤ 91.54	0.039	Reference
> 91.54		2.205 (1.04–4.672)
NLR		
≤ 1.36	0.040	Reference
> 1.36		2.621 (1.043–6.586)
SII		
≤ 914.59	0.126	Reference
> 914.59		1.65 (0.869–3.135)
LR		
≤ 37.3	0.071	Reference
> 37.3		0.427 (0.17–1.074)
Hb (g/L)		
≤ 107.1	0.141	Reference
> 107.1		0.585 (0.286–1.195)

Abbreviations: *SCC* squamous cell carcinoma, *PTV* planning target volume, *MD* maximum diameter, *BEDPD* prescribed dose recorded as biological effective dose, *BEDPTV*_*max*_ maximum biological effective dose in PTV, *PLR* platelet-to-lymphocyte ratio, *NLR* neutrocyte-to-lymphocyte ratio, *SII* platelet counts × neutrophil counts ÷ lymphocyte counts, *LR* lymphocyte ratio, *Hb* hemoglobin concentration

### Model construction

**Fig. 2:**
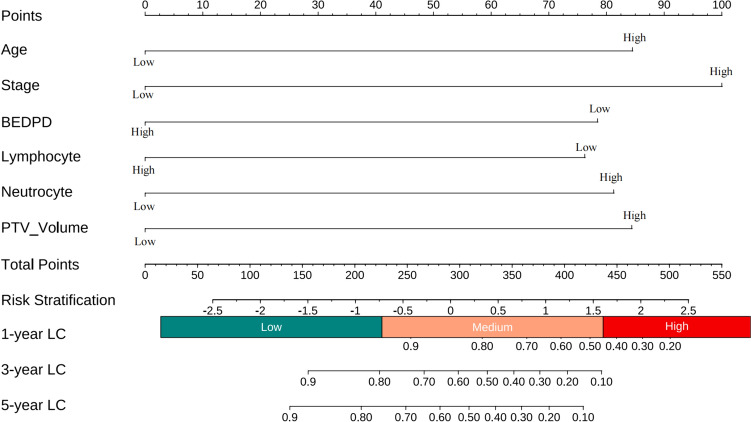
The nomogram for 1-year, 3-year and 5-year LC probability prediction. BEDPD = prescription dose recorded as BED, PTV = planning target volume, LC = local control

**Fig. 3:**
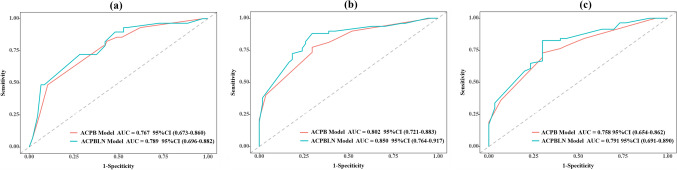
The 1-year, 3-year and 5-year ROC for the ACPB and ACPBLN models. **a** The 1-year ROC of the two models. **b** The 3-year ROC of the two models. **c** The 5-year ROC of the two models. ACPB model = model constructed by age, clinical stage, PTV volume, and BEDPD. ACPBLN model = model combined the lymphocyte count and neutrocyte count with the factors from the ACPB model

**Fig. 4:**
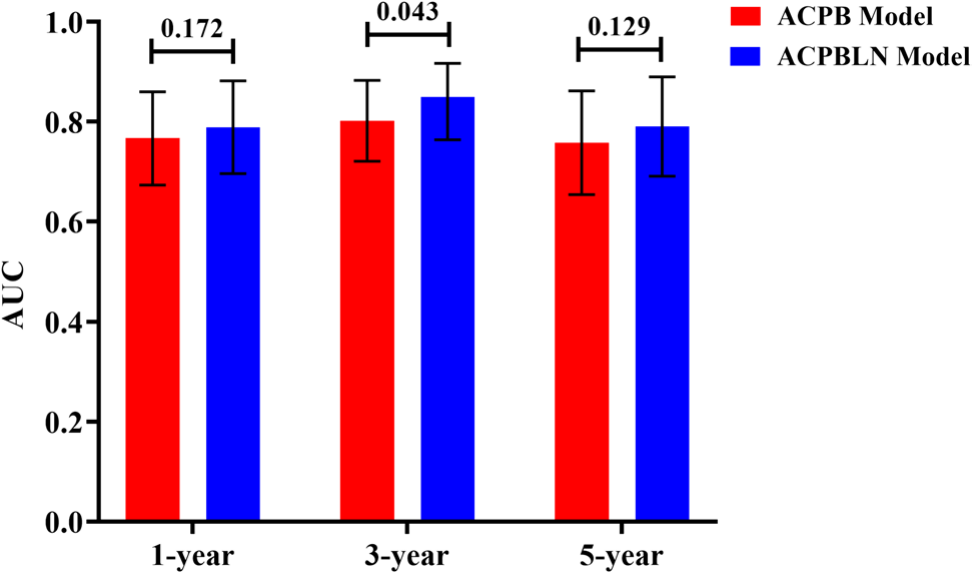
AUC value comparison for the ACPB and ACPBLN models. ACPB model = model constructed by age, clinical stage, PTV volume, and BEDPD. ACPBLN model = model combined the lymphocyte count and neutrocyte count with the factors from the ACPB model

**Fig. 5:**
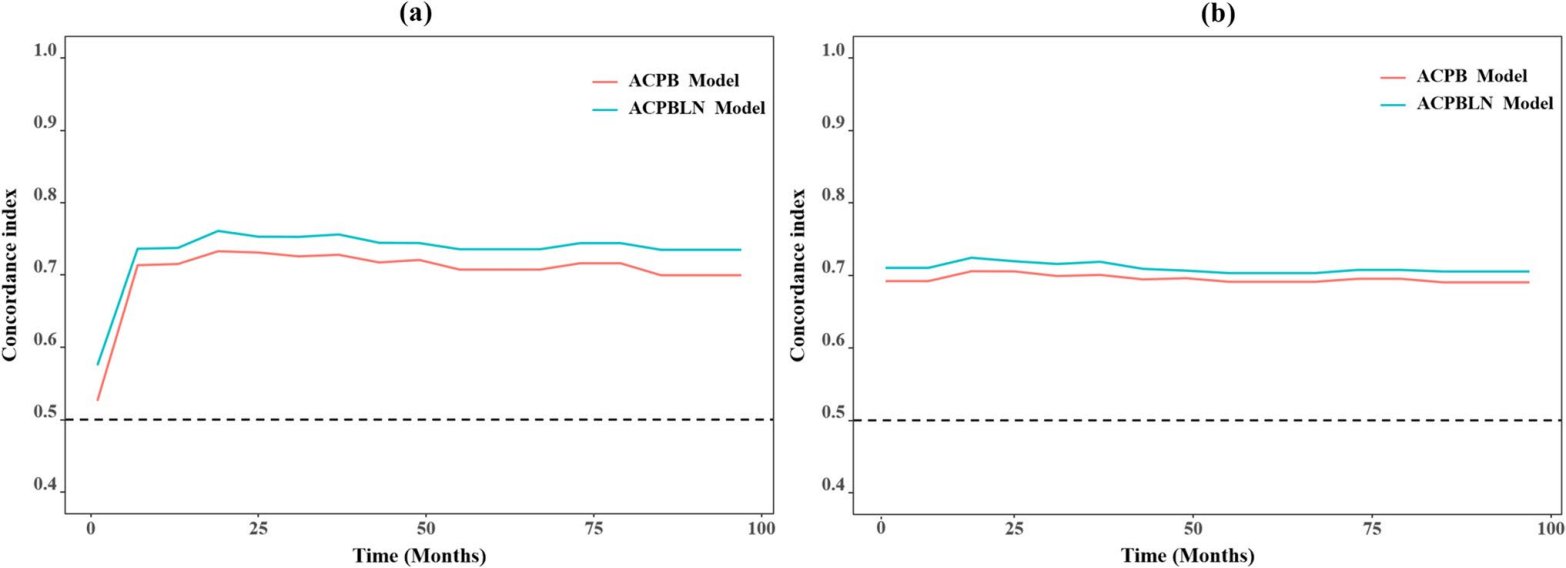
The time-dependent AUC and bootstrap-corrected time-dependent AUC for the ACPB and ACPBLN models. **a** Time-dependent AUC of the ACPB and ACPBLN models. **b** Bootstrap-corrected time-dependent AUC of the ACPB and ACPBLN models. ACPB model = model constructed by age, clinical stage, PTV volume, and BEDPD. ACPBLN model = model combined the lymphocyte count and neutrocyte count with the factors from the ACPB model

The nomogram for predicting 1-year, 3-year and 5-year LC probability was successfully constructed using the factors screened by the multivariate Cox regression analysis (Fig. 2). The 1-year, 3-year and 5-year ROC for the ACPB and ACPBLN models were shown in Fig. 3. Figure 4 displayed the comparison of the AUC values. The ACPBLN model showed improved performance in the 3-year AUC value compared to the ACPB model (*P* < 0.05), while the 1-year and 5-year AUC values were similar for both models(*P* > 0.05). The ACPBLN model had a lower AIC value (479.04 vs. 484.82) improved C-index and bootstrap-corrected C-index (0.745 vs. 0.719, *P* = 0.103) compared to the ACPB model. Table 2 listed the AIC value, C-index, and bootstrap-corrected C-index for both models. Additionally, Fig. 5a and Fig.5b showed the time-dependent AUC and bootstrap-corrected time-dependent AUC of the ACPB and ACPBLN models. The analysis of the time-dependent AUC also revealed that the ACPBLN model had better prognostic accuracy than the ACPB model.

**Table 2:** The AIC value and C-index of the ACPB and ACPBLN models

Variables	ACPB model	ACPBLN model
AIC	484.82	479.04
C-index (95% CI)	0.719 (0.660–0.778)	0.745 (0.684–0.806)
Bootstrap-corrected C-index (95% CI)	0.719 (0.658–0.774)	0.745 (0.673–0.799)

Abbreviations: *AIC* Akaike Information Criterion, *ACPB model* model constructed by age, clinical stage, PTV volume, and BEDPD, *ACPBLN model* model combined the lymphocyte count and neutrocyte count with the factors from the ACPB model

To demonstrate the discriminative capacity of the ACPBLN model, we used NRI and IDI values for further analysis. These values were used to compare the quantitative and the probability differences between the two models in predicting patients’ LC status correctly. The continuous NRI and IDI values of the ACPB and ACPBLN models at three time points (1-year, 3-year, and 5-year) were provided in Table 3. Compared to the ACPB model, the NRI of the ACPBLN model increased by 29.0%, 45.7%, and 29.7% at the 1-year, 3-year and 5-year time points, respectively. Similarly, the IDI improved by 4.6%, 6.1%, and 4.2% at the same time points.

**Table 3:** The NRI and IDI improvement of the ACPBLN model compared to the ACPB model

Variables	NRI% (95% CI)	IDI% (95% CI)	P^*^
1-year	29.0 (5.7–56.4)	4.6 (0.5–10.6)	0.028
3-year	45.7 (13.1–77.8)	6.1 (1.3–14.3)	0.004
5-year	29.7 (−1.8–66.4)	4.2 (0.0–13.6)	0.044

Abbreviations: *NRI* net reclassification index, *IDI* integrated discrimination improvement

^*^P value for IDI

**Fig. 6:**
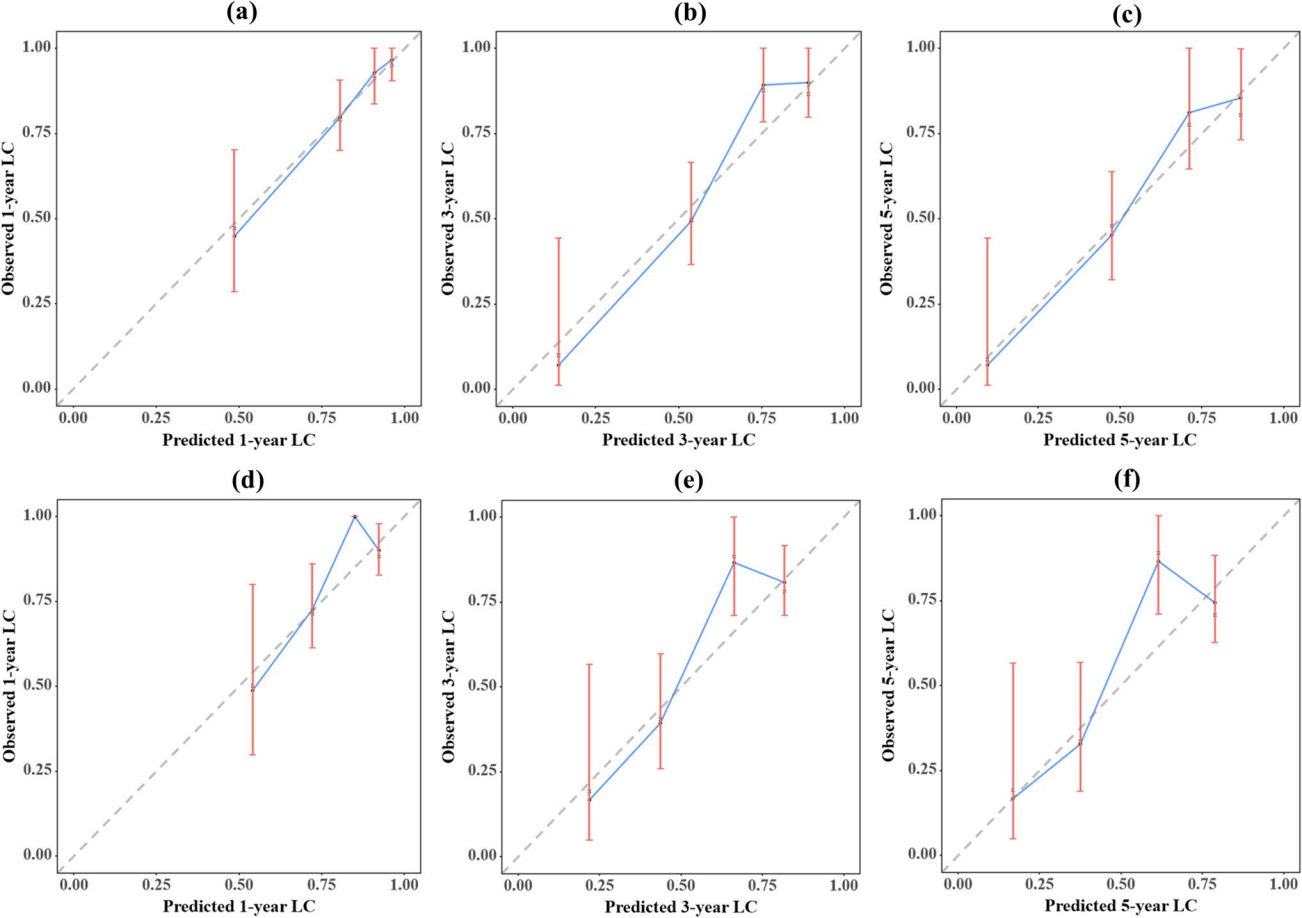
**a–c** Calibration plot for 1-year, 3-year and 5-year LC for the ACPBLN model. **d–f** Calibration plot for 1-year, 3-year and 5-year LC for the ACPB model. LC = local control

The calibration plots for the ACPBLN model and the ACPB model were displayed in Fig. 6, representing the 1-year, 3-year, and 5-year time points. Figure 6a, b, c showed the calibration plots for the ACPBLN model, while Fig. 6d, e, f showed the calibration plots for the ACPB model. These plots demonstrated that the predicted LC probabilities from both nomograms were in line with the actual observations at the 1-year, 3-year, and 5-year time points.

**Fig. 7:**
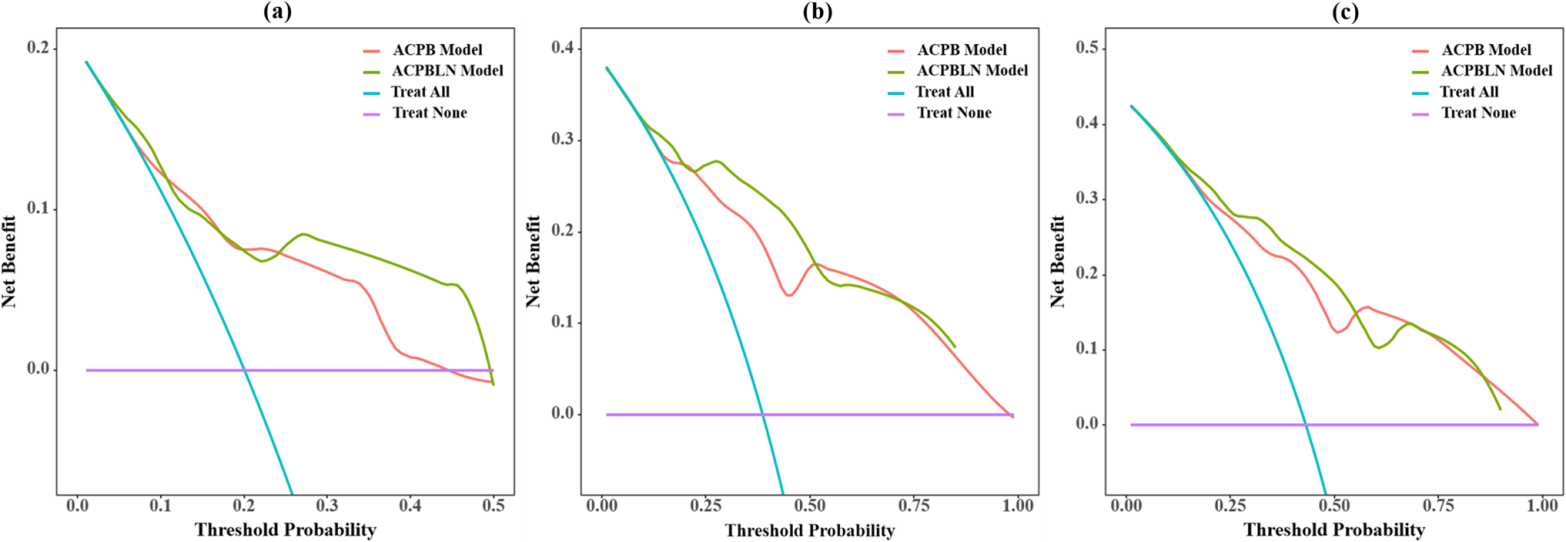
The DCA for the ACPB and ACPBLN models. **a** The DCA at 1-year time point. **b** The DCA at 3-year time point. **c** The DCA at 5-year time point. ACPB model = model constructed by age, clinical stage, PTV volume, and BEDPD. ACPBLN model = model combined the lymphocyte count and neutrocyte count with the factors from the ACPB model

The DCA of the two models at 1-year, 3-year and 5-year time points were displayed in Fig. 7. As shown in Fig. 7, the DCA curves indicated that the nomogram for the ACPBLN model had a higher overall net benefit than the ACPB model at the 1-year, 3-year, and 5-year time points across most threshold probabilities.

## Discussion

The study found that inflammation-related parameters, including the lymphocyte count and neutrocyte count, were independently correlated with LC in lung cancer patients treated with SBRT. Furthermore, we demonstrate that including these inflammation-related parameters contributes to improving the performance of the nomogram for LC prediction after SBRT. To our knowledge, this is the first study to demonstrate that including lymphocyte count and neutrocyte count improves the accuracy of LC prediction for lung cancer patients undergoing SBRT. We believe that the finding of the study have implications for LC prediction in lung cancer patients receiving SBRT.

Recently, there has been increasing attention on the prognostic role of inflammation-related factors in the era of immunotherapy. Several independent studies reported that these factors were associated OS in lung cancer patients treated with SBRT. Sebastian et al. discovered that a higher pre-treatment NLR was associated with inferior OS in SBRT-treated patients (Sebastian et al. 2019). Subsequently, this finding was confirmed in studies on single and multi-fraction SBRT for early-stage lung cancer (Dong et al. 2023; Huang et al. 2023; Kotha et al. 2021). However, there is limited evidence regarding the prognostic value of lymphocyte count and neutrocyte count for predicting LC after SBRT. We have found that a higher lymphocyte count or lower neutrocyte count contribute to an improved LC rate. These markers of inflammation and immune status can be easily, inexpensively, and repeatedly obtained, making them potentially applicable in routine clinical practice (Suzuki et al. 2019).

The finding that a higher lymphocyte count or lower neutrocyte count contributes to an improved LC rate is accordance with other publication in which the prognostic value of these factors is well described. Previous studies showed that a higher NLR was associated with inferior PFS and OS when using adjuvant immunotherapy for lung cancer (Bryant et al. 2022). Additionally, a recent study found that a higher NLR was significantly associated with poorer overall and progression-free survival, as well as lower response rates and clinical benefits after immune checkpoint inhibitors therapy across various types of cancer (Valero et al. 2021). Systemic inflammation has been shown to broadly influence tumor development and progression (Grivennikov et al. 2010). In fact, an elevated NLR indicates an increase in the absolute number of neutrophils and/or a decrease in the absolute number of lymphocytes. An elevated NLR may indicate neutrophil activation, which is linked to cancer progression, reduced effectiveness of immunotherapy, and unfavorable clinical outcomes in various cancers (Szczerba et al. 2019; Patel et al. 2018; Shaul and Fridlender 2019). Neutrophils has been reported to secrete tumor growth factors, cytokines, and chemokines, promoting angiogenesis (Grivennikov et al. 2010; Templeton et al. 2014). A recent study demonstrated that neutrophils in NSCLC inhibited anti-tumor immune responses by suppressing the cytotoxic activity of immune cells, particularly activated T cells (Kargl et al. 2017). On the other hand, a higher NLR also indicates lymphocyte depletion, which may be associated with inadequate anti-tumoral immune responses (Qin et al. 2017). Lymphocytes play a vital role in the human immune system and are crucial for the immune response against cancer. When the lymphocyte count decreases, the effectiveness of the immune system in fighting tumors is reduced, leading to accelerated tumor growth (Lin et al. 2006). This analysis helps to explain why a decrease in neutrophils or an increase in lymphocytes can improve the LC rate of lung tumors. However, additional research is needed to confirm the relationship between these two inflammation-related factors and LC, and to understand the immune mechanisms involved.

Statistical analysis of data plays an important role in establishing prediction models. In this study, we transformed continuous variables into categorical variables to explore the relationship between clinical, dosimetric and inflammation-related factors and LC. Although this approach may result in some loss of useful information, it helps to identify optimal cut-off points and simplify the model interpretation and application in clinical practice. While this approach may be somewhat controversial, there are three key facts that demonstrate the successful establishment of the model in this study. First, the 95% CI for the factors are within normal range. Second, the selected factors have clinical interpretability. Third, some of the screened factors agree well with results of existing literature.

However, the study has some limitations. Firstly, retrospective studies were inevitably subject to selection bias. Secondly, the study has limited sample size. The study does not satisfy the 10 events per variable (EPV) rule (Riley et al. 2020). Therefore, the robustness of the conclusion should be further confirmed in external institutions. Thirdly, there was a risk of competing mortality events due to dead cases without local recurrence in the study. And the competing mortality events might partially weaken our result. Lastly, it is wonder whether the inclusion of both primary and metastatic lung cancer patients for LC model construction may weaken our result. However, Guckenberger et al. reported that the tumor LC in SBRT did not vary between primary lung cancer and lung metastases (Guckenberger et al. 2016). The similar conclusion was also obtained in this study when performing the univariate analysis. Therefore, we believe that the heterogeneity of tumor origin will not affect the results of this study.

## Conclusion

The inflammation-related parameters were independently correlated with LC in lung cancer patients treated with SBRT. Including these parameters improved the performance of the nomogram for predicting LC.

## Data Availability

No datasets were generated or analysed during the current study.
